# Simultaneous rotary and linear displacement sensor based on soft pneumatic sensing chambers

**DOI:** 10.1038/s41598-024-59168-3

**Published:** 2024-04-09

**Authors:** Alireza Ghaffari, Yousef Hojjat

**Affiliations:** Department of Mechanical Engineering, Tarbiat Modares University, Tehran, Iran

**Keywords:** Mechanical engineering, Electrical and electronic engineering

## Abstract

Specific industrial or research applications necessitate specialized displacement measurement conditions, thereby driving researchers to innovate sensors based on novel operating principles. One such challenging condition is the prevalence of strong electromagnetic waves, which precludes using any sensor with a metallic structure or one that operates on electrical measurement principles. Additionally, space constraints in applications requiring multidimensional displacement measurements mandate the development of sensors capable of measuring displacements simultaneously in multiple directions. This paper introduces a novel soft sensor designed to simultaneously measure linear and rotational displacements using Soft Pneumatic Sensing Chambers (SPSCs). This sensor is unique in its ability to measure both linear and rotational movements and, due to its Electro-Magnetic Compatibility (EMC) and compact size, is suitable for environments with significant electromagnetic interference and spatial constraints. Furthermore, its flexibility makes it appropriate for body-interacting applications. The Abaqus software was employed to optimize the operating parameters. Subsequently, a laboratory setup was assembled, and the sensor's performance was assessed using two calibration methods: mathematical modeling and machine learning. According to the machine learning method, the accuracy in the linear and rotational directions was 0.49 mm and 5.4°, while the Root Mean Square Error (RMSE) was 0.05mm and 0.48°, respectively.

## Introduction

The challenge of measuring multi-dimensional displacements has been a longstanding issue in the field of instrumentation. Various methods have been devised to address this need, most involving the separation of movements along different axes. This approach, however, increases both the required space and the complexity of the motion mechanisms. Conversely, recent research has yielded a limited number of methods for simultaneously measuring different degrees of freedom. These methods are primarily categorized into three groups: capacitive, magnetic, or optical^1–5^.

Rotary-linear movement is a type of multi-degree-of-freedom movement encountered in various applications, making its measurement crucial for developing closed-loop systems. Applications requiring the measurement of rotary and linear displacements include capping machines, pick-and-place tasks in production lines, needle insertion robots for biopsy procedures, laboratory processes such as Polymerase Chain Reaction (PCR) for sample displacement, handheld football game controllers, and remote control systems^6–9^. Gao et al. successfully measured rotary-linear movements using a surface encoder comprising a microstructured surface and a system for processing the light reflected from this surface^10^. Laser interferometry, another method, accurately measures two-dimensional movements despite its rotational range limitations^11^.

Image processing has also been employed to measure this movement, but it necessitates a high processing volume and space for installing the image sensors^6^. Anandan et al., as well as Kumar et al., managed to measure an axis's linear and rotational movements by measuring the capacity of 4 and 6 capacitors, respectively, and deriving its mathematical mode^7,12^. In another study, researchers managed to measure the simultaneous linear and rotational motions of an axis by utilizing triboelectric and electrostatic induction effects^13^. Among the weaknesses of these researches, we can point out their electromagnetic incompatibility due to their metallic structure and electrical measurement nature. The optic method, nearly the only available Electro-Magnetic Compatible (EMC) displacement measurement technique, has been employed in single-degree-of-freedom measurements^14^. Also, the mentioned methods are not electrically isolated and could not be used in explosive environments.

To address these weaknesses, the soft measurement technique has been employed in this research. The surge of interest in soft robotics in recent years has led to further advancements in the field of soft sensors. Before this development, the Force-Sensing Resistor (FSR) was the only soft sensor that had garnered significant research attention and commercialized. The popularity of this sensor can be ascribed to its flexibility and thinness, attributes absent in sensors with rigid mechanical structures. Soft sensors can be fabricated using more readily available and expedited methods, such as 3D printing and silicon rubber molding, and also exhibit resilience in diverse environments^15,16^. A notable characteristic of these sensors is that, unlike traditional sensors, they do not constrain the degrees of freedom of the objects being measured, making them suitable for measuring displacements with multiple degrees of freedom^17–19^. One such method, which has been proven effective for measuring movements with one degree of freedom, involves the use of Soft Pneumatic Sensing Chambers (SPSCs)^20–23^. This technique measures displacements by detecting changes in the air pressure trapped within flexible chambers.

In a previous article, we successfully introduced a Soft Pneumatic Rotary Encoder (SPRE) based on this measurement method^24^. The present article makes an effort to design, optimize, and fabricate a soft sensor for measuring simultaneous linear and rotational movements using a specific arrangement of SPSCs. Through this research, we were able to generalize the idea of SPRE to more complex and challenging multi-dimensional movements. This represents the first instance where the efficacy of SPSCs in multidimensional measurements is proven. Unique features of this sensor include electro-magnetic compatibility and electrical isolation from the object being measured, attributes that are a consequence of its non-electrical measurement nature and entirely non-metallic structure. As a result, this sensor is the only available rotary-linear sensor that can be employed without special shielding conditions or Electro-Magnetic interference (EMI) in MR-guided robots^25^. Owing to its ability to measure degrees of freedom simultaneously, this sensor requires minimal space, making it suitable for the confined space of MRI bore and any other applications with space limitations.

Additionally, the mechanical movement mechanism of the axes is simple and backlashless. The passivity of this measurement method allows its usage in hazardous environments, such as explosive atmospheres, and in applications involving direct contact with humans, such as rehabilitation equipment. On the other hand, in single-axis displacement measurement applications, this sensor has the capability to tolerate unwanted linear movements of the rotary actuator and unwanted rotary displacements of the linear actuator.

Calibrating multi-degree-of-freedom sensors presents another challenge: dealing with Multiple-Input Multiple-Output (MIMO) systems. MIMO systems can be calibrated by extracting mathematical equations or employing machine learning algorithms^23^. In a study referenced as^26^, the capacitance of 8 dielectric elastomers was utilized to compute the displacement of a 5-degree-of-freedom actuator. This study demonstrated that MIMO sensors could be calibrated using the Support Vector Machine (SVM), one of the machine learning algorithms. Kawato et al., by creating a system consisting of three two-axis Hall effect sensors and using the Gaussian Least-Squares Differential-correction (GLSDC) method to solve its mathematical model, introduced a sensor with three degrees of freedom^3^. In this article, both mathematical modeling and machine learning methods are evaluated.

The structure of the article is as follows: First, the concept design, functional mechanism, and sensor optimization using the finite element method are discussed. Then, the fabrication process of the sensor and its test setup is explained. Finally, the test results are stated and analyzed.

## Materials and methods

### Sensor concept and working principle

**Figure 1:**
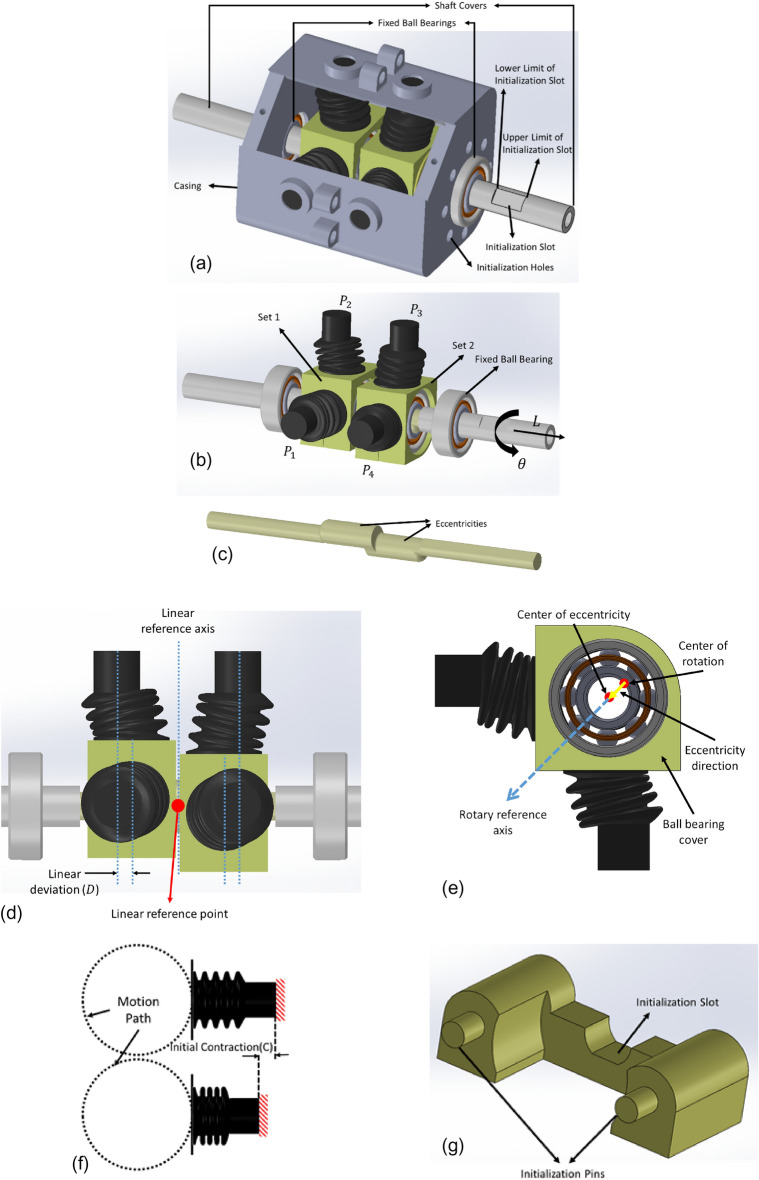
Concept and parameters introduction: (**a**) schematic of the sensor representing the main concept, (**b**) chambers arrangement and $L-\theta$ direction, (**c**) design of eccentricities, (**d**) linear deviation (D) and references, (**e**) eccentricity direction and rotary reference axis, (**f**) Initial contraction parameter (C), (**g**) the designed gauge which holds the sets on their home positions to connect to the ambient pressure during the initialization process.

In the SPRE study, the pressure inside of the SPSCs was employed to calculate the rotation of an eccentric shaft^24^. To overcome the decreased measurement sensitivity of each SPSC at the start and end of the measurement range, two SPSCs with a 90-degree phase difference were utilized. This approach was replicated in the present study, albeit with two sets of SPSCs (Fig. 1a) and (Fig. 1b). A 90-degree rotational phase difference between the two sets was considered to ensure the sensitivity of the sensor all over the working range. Rather than applying this phase difference to the SPSCs' placement, it was applied to the shaft by considering another eccentric axis (Fig. 1c). The axis of symmetry shown in Fig. 1d is considered as the reference axis in linear displacements, and the distance of this axis from reference point, which is the middle point of the shaft, represents the value of L. The deviation of each set from its straight configuration is called linear deviation (D), and its value when L = 0 is the initial linear deviation $({D}_{0})$. If ${D}_{0}$ is zero, the two sets will not have any phase difference in the linear direction and they will be parallel, as a result, the linear displacement measurement becomes impossible. Therefore, it is necessary to determine a non-zero value for ${D}_{0}$. The direction of eccentricity and rotary reference axis are depicted in Fig. 1e. The $\theta$ parameter is calculated based on the angle between the direction of eccentricity and the reference rotary direction in set 1. To prevent any deviation and backlash in ball bearings, each set incorporated two rows of bearings. Consistent with our previous research, we employed the sbl15 model suction cup by Airbest as the SPSC^24^. This Nitrile Butadiene Rubber (NBR) suction cup features an active volume with a 15.5mm diameter and a 13.7 mm length. Its compact size and suitable number of steps in its active length informed this choice.

Another influential parameter is the initial contraction (*C*), representing the SPSC's contraction amount at its maximum contraction (Fig. 1f). For simplification, the SPSC-connected wall’s motion path is presumed to be circular. The impacts of parameters ${D}_{0}$ and *C* will be subsequently assessed via simulation in Section "Simulation".

The sensor activation process commences with an initialization procedure, during which each set must return to its home position ($D=0$ and $\theta =0^\circ$ for set1 and $\theta =90^\circ$ for set2) and connect to the ambient air pressure. This ensures the repeatability of sensor calibration. A slot has been considered on the shaft to align the sets with their home positions, and two holes have been considered on the sensor casing. Additionally, a gauge (Fig. 1g) has been designed. When the initialization pins coincide with the casing holes and the gauge makes contact with the initialization slot on the shaft, it aligns the sets with their reference angles. Moreover, by moving the shaft to contact the upper and lower boundaries of the initialization slot with the gauge, each set returns to its $D=0$ point.

To eliminate the effect of temperature, a temperature neutralizer tube was placed inside a sheath along with other tubes. This closed-end tube continues inside the sensor and its pressure is constantly measured. This tube is connected to the ambient pressure with the help of a valve during the initial setup process. Because the temperature of this tube is the same as the temperature of the air trapped in other tubes, the result of dividing the pressure of SPSCs by the pressure of this tube will result in pressure ratio parameters (${R}_{1}, {R}_{2}, {R}_{3}, {R}_{4}$) independent of temperature. The R parameters are expressed in percentage.

### Simulation

The Fluid cavity tool of Abaqus software was utilized for the finite element simulation of the sensor. *C* and *D*_*0*_ were applied to the initialization procedure through a displacement boundary condition. During the initialization process, wherever needed, the pressure was set to zero using the fluid cavity pressure boundary condition. A reference point was defined on the shaft and coupled to the shaft. Theta and El boundary conditions were applied to this point. Also, another point was defined on the eccentric axis, and by coupling it to the bearing cover, the role of the bearing was implemented. Due to the possibility of contact between the steps of SPSCs, self-contact interaction was applied to them. Tubes reduce the range of sensor pressure changes. In fact, pressure changes are attenuated by the ratio of SPSC volume to the total volume of trapped air. Tubes have been omitted in the simulation, so to apply this effect, the attenuation factor was multiplied by the ambient pressure. At first, a simulation was carried out to calculate the volume of SPSCs at the moment of connection to atmospheric pressure during the initialization process for each combination of *C* and *D*_0_. Considering the volume of the tubes, which is $3534 {{\text{mm}}}^{3}$, the range of attenuation factor was between 0.21 and 0.23, according to the different combinations of *C* and *D*_0_. The volume of the active portion of the SPSC without applying any deformation is about $1330{{\text{mm}}}^{3}$. This simulation was done assuming an ideal gas with a molar mass of $28.8 {\text{g}}$ at $25^\circ{\rm C}$.

The sensor's rotational and linear motion ranges were deemed infinite and ± 6.5 mm, respectively. Due to symmetry, the simulation was conducted for one of the sets within the 0°–180° range. *C*, *D*_0_ parameters should be selected so that the difference between the highest and lowest pressures observed during the working interval ($\Delta P$) is maximized. On the other hand, to keep the sensor fixed, we need a holding torque and holding force, and their maximum should be minimized.

**Figure 2:**
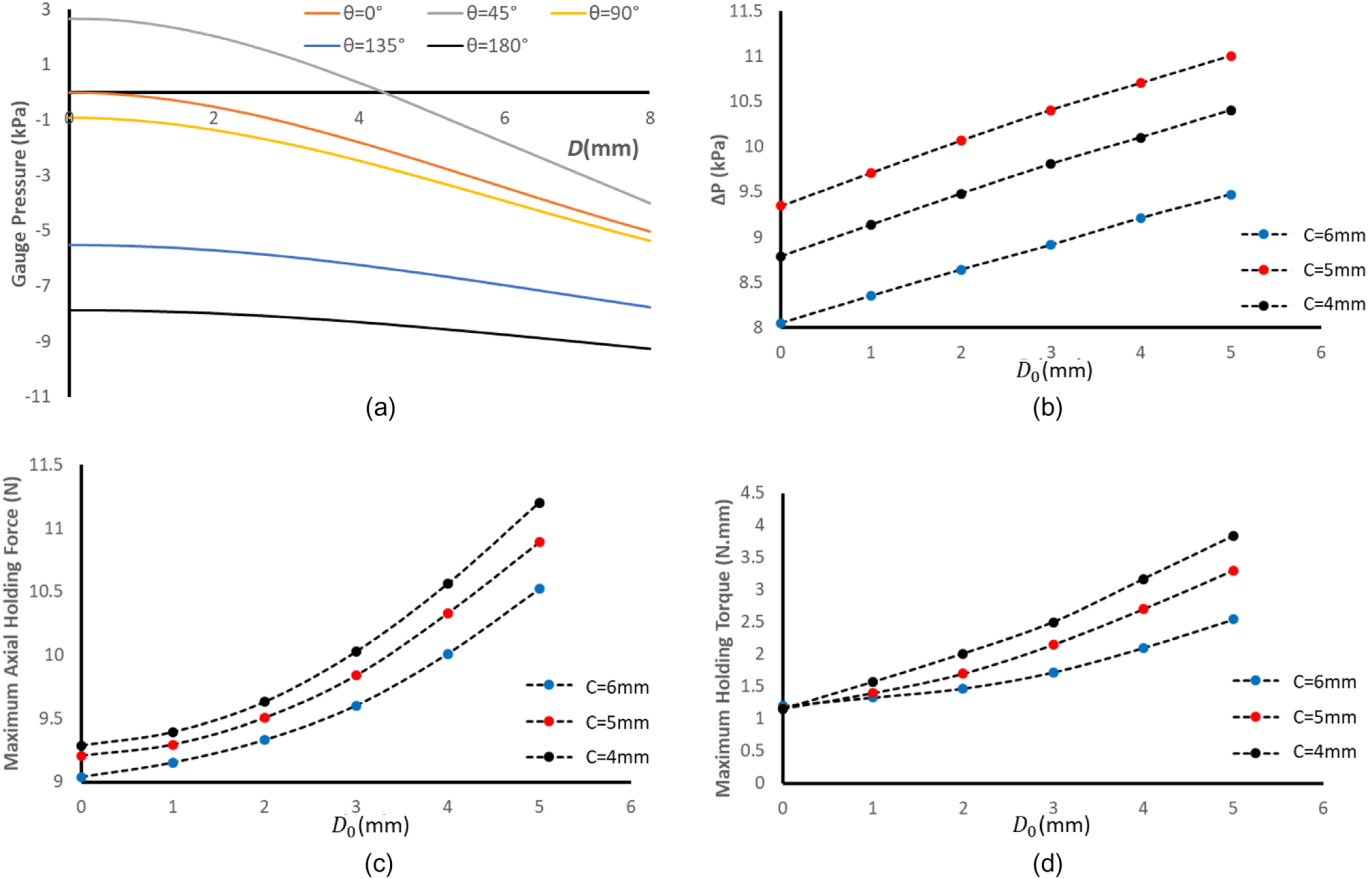
Results of the simulation (**a**) the effects of $D$ on gauge pressure in chamber 1 (**b**), (**c**), (**d**) the effects D_0_ and C on the amplitude of the pressure change ($\Delta P$), maximum holding force and maximum holding torque of the sensor.

Figure 2a illustrates the pressure in chamber number 1, resulting from changing *D*. The $D<2$ region is critical due to the low rate of pressure changes with *D*. Therefore, the D parameter should be designed in such a way that both sets do not locate in this region at the same time. This requirement is fulfilled if ${D}_{0}\ge 2{\text{mm}}$ is stipulated.

Based on Fig. 2b, parameter C has an optimal value at 5mm where the pressure changes are maximum. In other words, this parameter causes the SPC working interval to be selected in such a way that the volume changes are maximum. The maximum amount of pressure will occur at $D=0$. Therefore, the maximum pressure observed in the entire working range is independent of the ${D}_{0}$ value. Increasing the value of ${D}_{0}$ will cause the SPSCs to be in the range with more tension, and we will face less pressures in the system. This increase in the pressure range is shown in the Fig. 2b, which behaves almost linearly with ${D}_{0}$. An increase in ${D}_{0}$ and a decrease in C increase the holding force and torque due to the increase in the extension of the SPSCs. The exponential form of these curves can be due to the exponential form present in the force–displacement diagrams of the SPSCs.

Per the graphs in Fig. 2b–d, if the design objective is to augment the sensor's pressure variation range, a *C* value of 5 mm should be adopted. However, if the goal is to minimize the holding torque and holding force, a *C* value of 6mm is preferable. In this study, *C* = 5 was selected as it represents a compromise between the torque and force parameters and maximizes ∆P. Since the influence of elevating the *D*_0_ parameter on the ∆*P* value is marginal (Fig. 2b), but it considerably impacts force and particularly torque, the *D*_0_ value was set at 2mm in this study to satisfy the condition ${D}_{0}\ge 2{\text{mm}}$.

### Fabrication and testing

**Figure 3:**
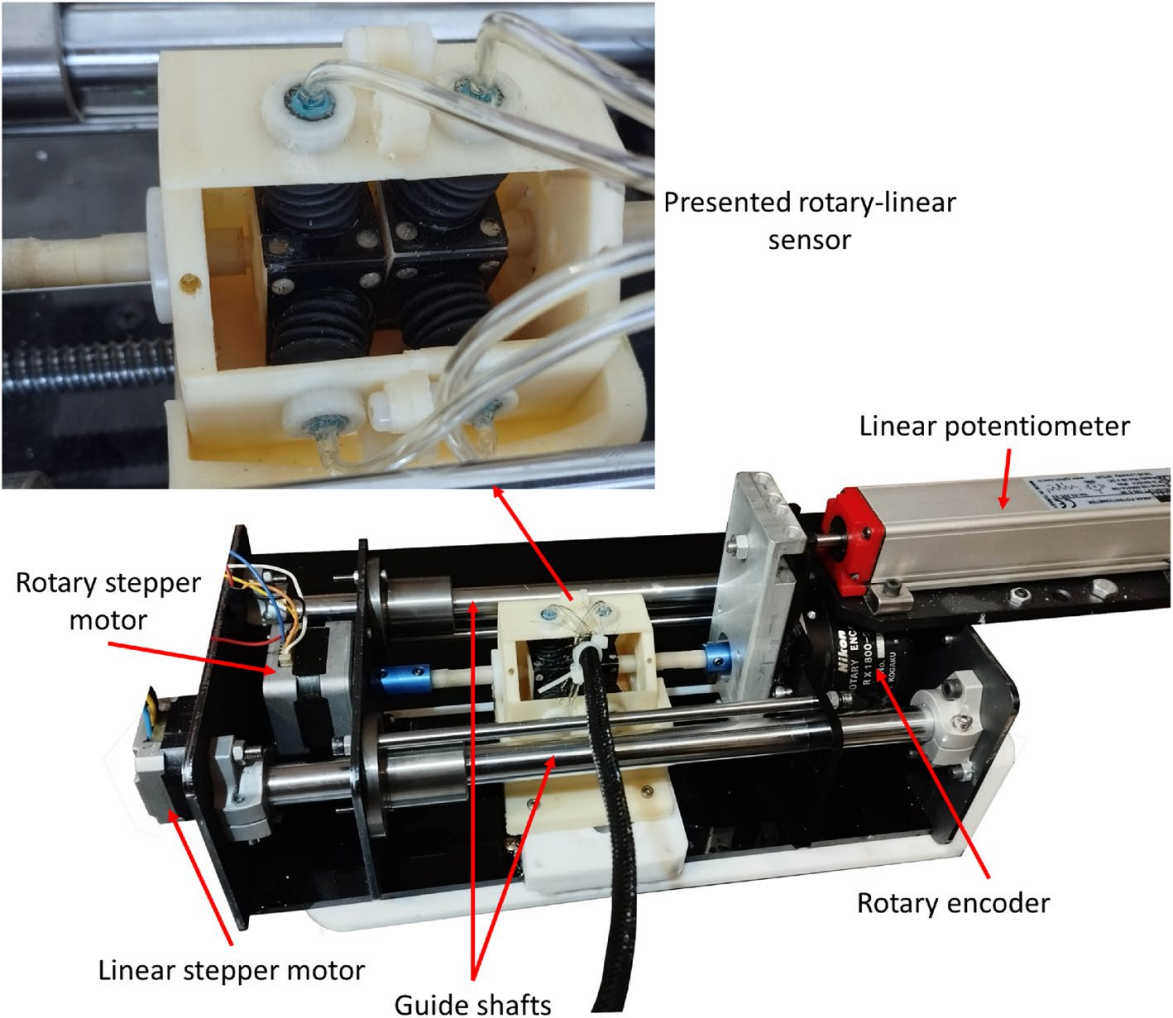
Presented sensor and its test setup including two degrees of freedom displacement mechanism and displacement measurement sensors.

The fabricated sensor is depicted in Fig. 3. The casing and shaft were fabricated using 3D-printed Acrylonitrile Butadiene Styrene (ABS). Since the shaft is subjected to wear from linear movements, and Polyamide (PA) offers superior wear resistance compared to ABS, the shaft cover was 3D-printed from PA. A Polyurethane (PU) tube, with internal and external diameters of 1.5 mm and 3 mm, respectively, was employed to connect the SPSCs to the DAQ unit. Cyanoacrylate glue was used to affix the ball bearing cover to the SPSC, the SPSC to the casing, the tube to the SPSC, and the tube to the pressure sensor. Additionally, all the bearings utilized were made of polymer. The specifications of the fabricated sensor are detailed in Table 1.

**Table 1:** Dimensional details of the sensor.

Parameter	Value
Width * Length * Height	47 mm * 47 mm * 63 mm
Eccentricity (e)	2 mm
Initial contraction (C)	5 mm
Initial linear deflection (${D}_{0}$)	2 mm
Tube length	2000 mm
Tube outer diameter	3 mm
Tube inner diameter	1.5 mm
Output shaft diameter	8 mm

Five BMP280 sensors manufactured by Bosch were used to measure the pressure and temperature of four SPSCs and the ambient air. During the initialization procedure, pneumatic valves were used to connect the chambers to the ambient air. To assess the sensor's performance, a laboratory setup comprising two stepper motors designed to generate linear and rotary movements was assembled (Fig. 3). The rotational movements generated by one of the motors were converted into linear displacement via a 3 mm pitch lead screw. Additionally, a Nikon RX1800 rotary encoder, with 7200 steps per revolution, and an Opkon RTL-100 linear potentiometer were used to measure the rotary and linear movements, respectively. The pressure sensors data were transmitted to an Arduino Mega 2560 microcontroller via an SPI interface. This microcontroller was also employed to generate the rotational pulses of the stepper motor drivers and read the position sensors. The testing procedure involved positioning the sensor's linear position at 100 µm intervals and executing a full cycle of back-and-forth rotation in 1.8° steps to measure the SPSCs' pressure values. The sensor was evaluated in 5 complete cycles of longitudinal movements for three days. Meanwhile, in order to check the effectiveness of the presented method in compensating the effect of temperature, the ambient temperature was changed in the temperature range of 17–28 °C.

## Results and discussion

**Figure 4:**
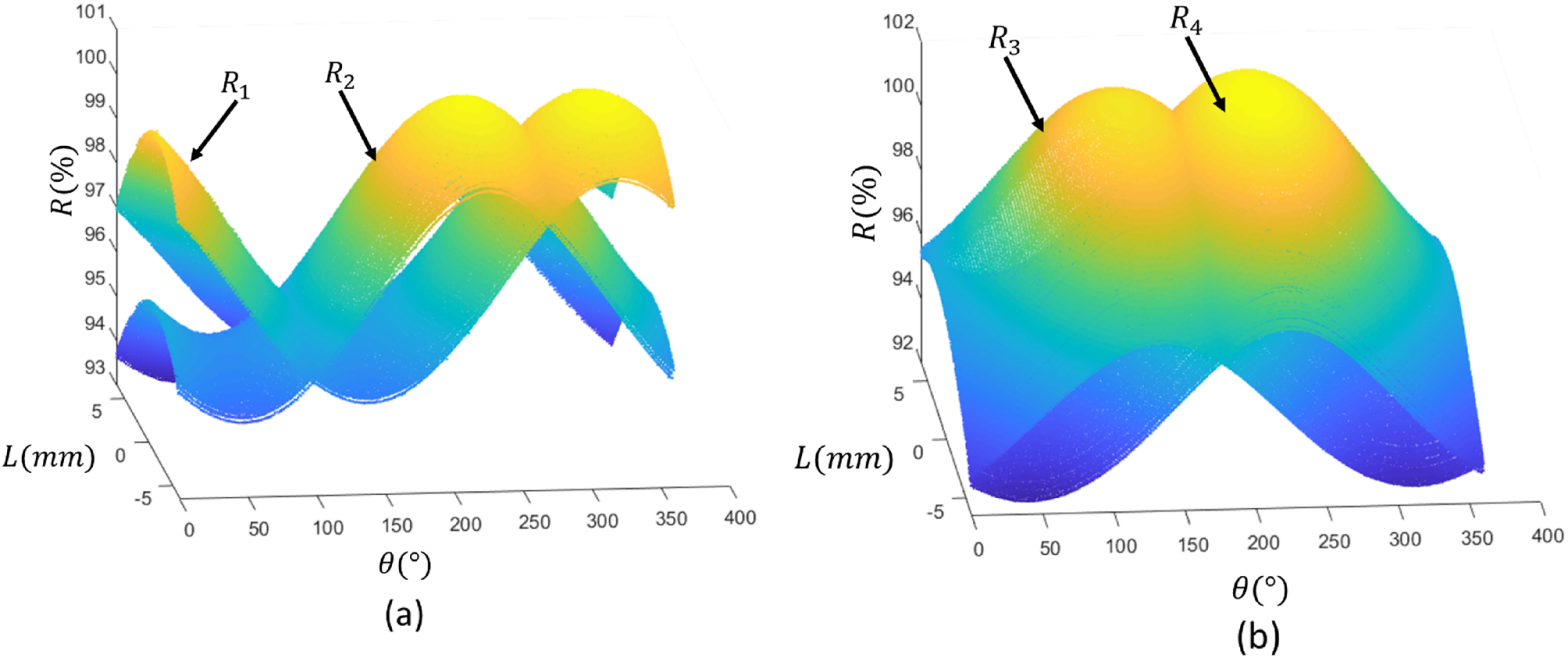
Results of the experimental tests (**a**) R1 and R2, (**b**) R3 and R4.

The R charts derived from the experimental tests on the fabricated prototype are illustrated in Fig. 4. The range of R ratio changes for SPSCs 1 and 2 is approximately $7$ For SPSCs 3 and 4, it is about $8.5$. This disparity is attributed to the pressure range observed in the two sets, which is approximately 6 kPa for the SPSCs in the first set and about 8.9 kPa for the second set. This parameter was 9.8 kPa in the simulations, which closely aligns with the measured values in the second set. The sole factor that could account for the discrepancy between the simulation and the values obtained in set 2 is the errors introduced during manual assembly. These errors could encompass inaccuracies in part fabrication due to the limited precision of the 3D printer and errors in adhesive application.

### Sensor calibration

Before presenting calibration models, the uniqueness of each ($\theta ,L$) position for the possible combination of (${R}_{1},{R}_{2},{R}_{3},{R}_{4}$) input values should be examined. For this purpose, 1 million pairs of positions were randomly selected. Their R distance and spatial distance for each pair, such as m and n, were calculated using Eqs. 1 and 2. These distance parameters have no physical meaning. In order to match the scale of the linear and rotary distances, they are normalized in Eq. 2.

1 $$\sqrt{{\left({R}_{1}^{m}-{R}_{1}^{n}\right)}^{2}+{\left({R}_{2}^{m}-{R}_{2}^{n}\right)}^{2}+{\left({R}_{3}^{m}-{R}_{3}^{n}\right)}^{2}+{\left({R}_{4}^{m}-{R}_{4}^{n}\right)}^{2}}$$

2 $$\sqrt{{\left(\frac{{L}^{m}-{L}^{n}}{13}\right)}^{2}+{\left(\frac{{\text{min}}(\left|{\theta }^{m}-{\theta }^{n}\right|,360-\left|{\theta }^{m}-{\theta }^{n}\right|)}{360}\right)}^{2}}$$

**Figure 5:**
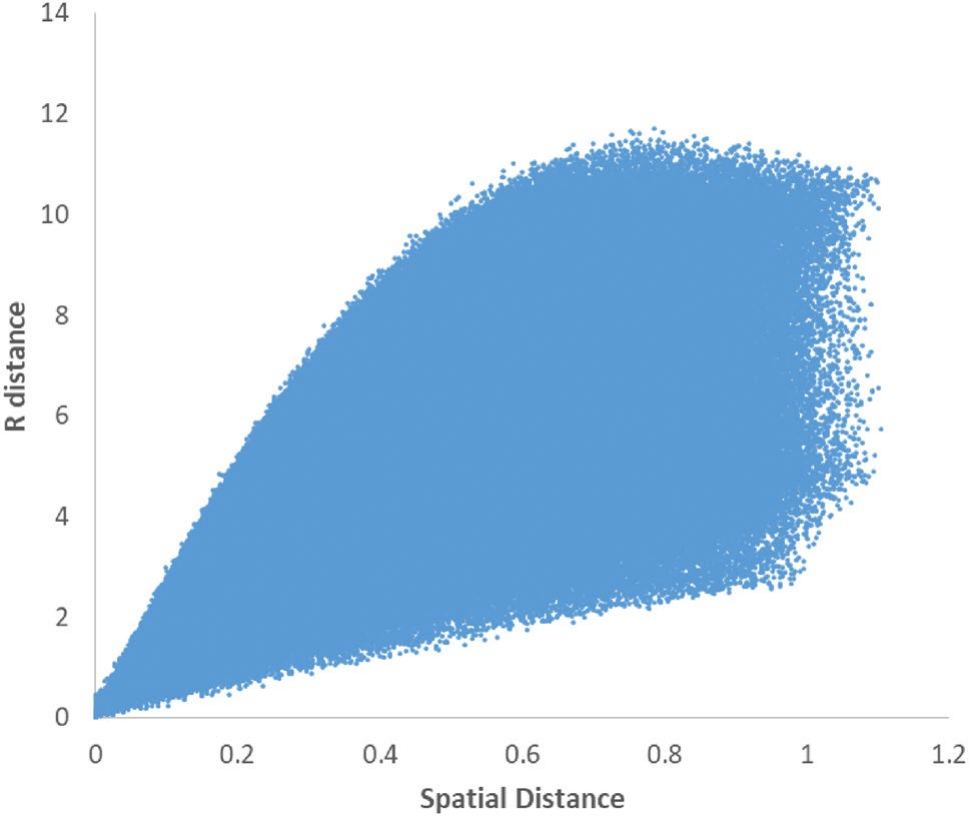
R distance against spatial distance values calculated for 1 million pairs of data points.

In order to ensure uniqueness, it is necessary for the data points to have no intersection with the horizontal axis except at the origin. As depicted in Fig. 5, this criterion is almost met by the data points depicted in this diagram.

**Figure 6:**
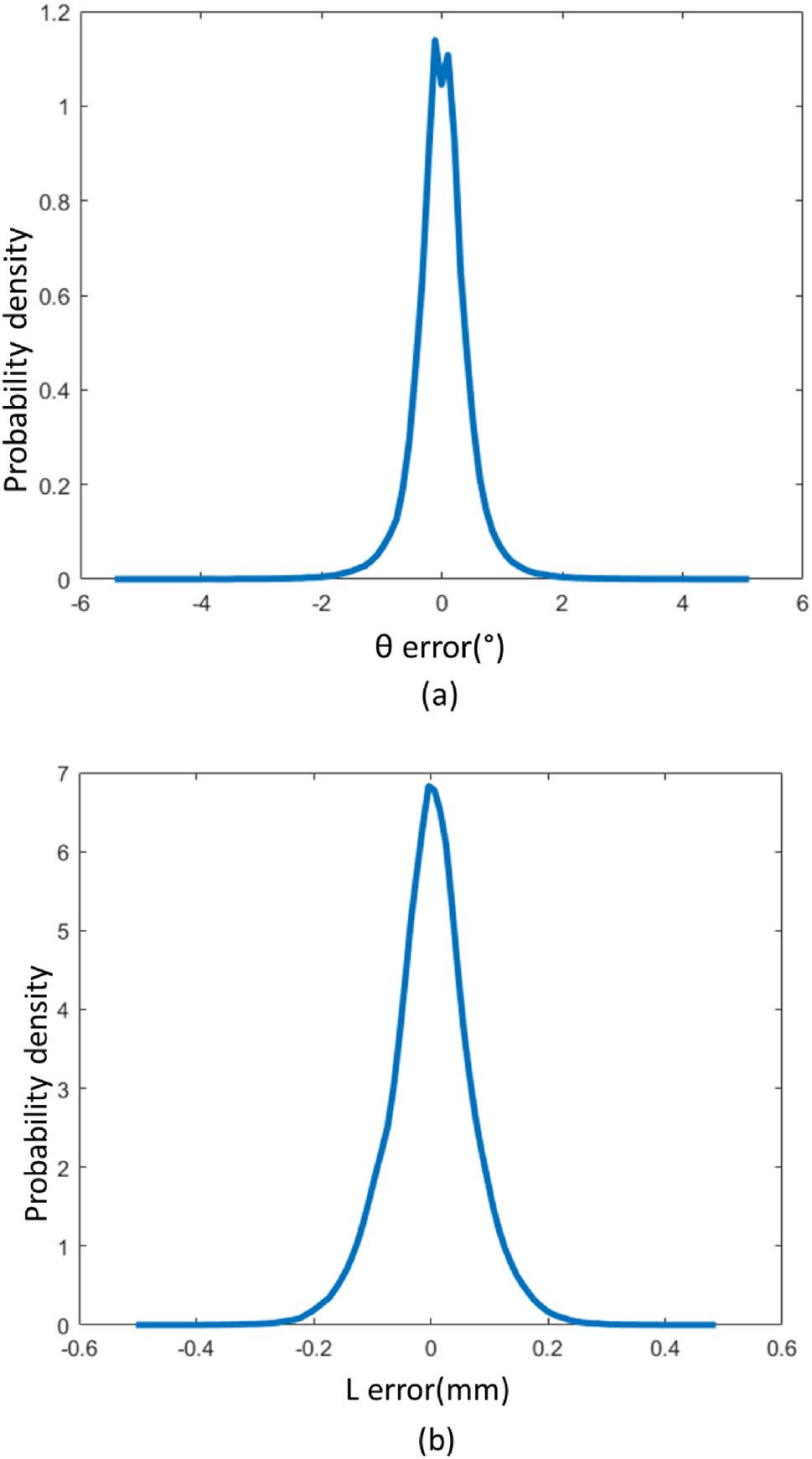
Error probability density (**a**) Rotary error, (**b**) Linear error.

The first approach employed for displacement calculation in this study is the Random Forest algorithm, one of the machine learning (ML) methods used for classification and regression. Random forest is an accurate algorithm that is robust to outliers and data noise. The Python programming language was used to implement the random forest regressor algorithm of the Sklearn library. Because data quality is highly important in machine learning, multiple actions have been taken to cleanse the dataset. The first phase entails configuring the pressure sensor, which transmits the pressure value after oversampling 16 times and IIR filtering with the coefficient of 2 in order to reduce noise and short-term fluctuations. In each position, pressure data were recorded two times in order to allow the model to learn the noise that might be associated with the measurement of the same position. Due to the discontinuity between the data at the start and end of the 0–360-degree measurement range and the inability to identify the periodic feature, the angle was substituted with its sine and cosine. After computing the R values in Excel software and eliminating wrong data received from serial port due to the high baud rate, the dataset was processed to the training phase. Moreover, the linear displacement data were normalized. Therefore, this algorithm maps four R values as inputs to three output features: sine and cosine of $\theta$ and the normalized value of $L$. The solution process involves dividing all the collected data, approximately 870 K, into two parts: 435 K for training and 435 K for test. The splitting is done randomly by “train_test_split” function of the “scikit_learn” library. We should determine the number of decision trees and the height of each tree in the algorithm, which are specified by the parameters n_estimators and max_depth, respectively. Increasing n_estimators and max_depth parameters more than 150 and 50 was found to be ineffective. The error probability densities resulting from “ksdensity” function of Matlab software are illustrated in Fig. 6. Characteristics of the proposed sensor with presented calibration methods are summarized in Table 2.

**Table 2:** Characteristics of the prototype sensor.

Calibration method	Measurement type	Accuracy	RMSE	Resolution
MM	Rotary	$5.6^\circ$	$1.4^\circ$	$0.06^\circ$
	Linear	0.8 mm	0.16 mm	0.016 mm
ML	Rotary	$5.4^\circ$	$0.48^\circ$	$0.06^\circ$
	Linear	0.49 mm	0.05 mm	0.011 mm

**Figure 7:**
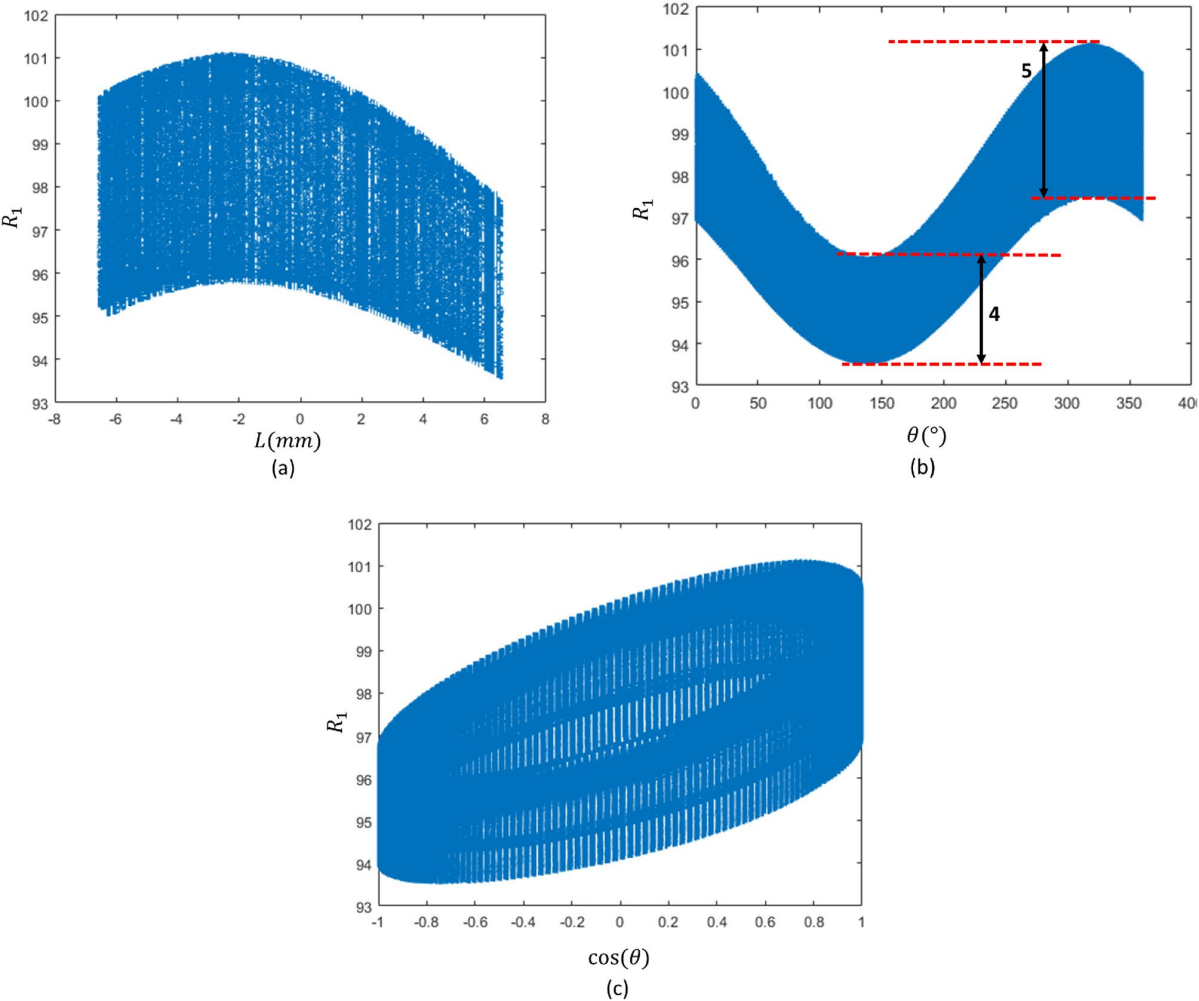
R1 diagram (**a**) in R1-L plane, (**b**) in R1-θ plane, (**c**) in R1-cos(θ) plane.

In this research, an alternative means of calculating displacement involves using mathematical modeling (MM). Developing a mathematic model using analytical relationships for soft sensors is often unfeasible due to their non-linear behavior and complex deformation. A viable alternative for calibrating these sensors is to employ regression methods to derive a model without resorting to mechanical deformation relationships. To determine the transfer function, for instance, we analyzed the changes of R1 in R1-θ, R1-L, and R1-cos(θ) planes (Fig. 7).

As observed in Fig. 7b and c, with moving along L, the modulated cosine graph on R1 also shifts. This shift is assumed to be a third-degree polynomial. Conversely, when moving along the L direction, the amplitude of the waves initially increases and then decreases. Then, the amplitude of these cosine curves also fluctuates with L and is postulated to be of the third degree. Consequently, an equation of the following form was contemplated for this diagram:

3 $${R}_{1}={c}_{1}{L}^{3}+{c}_{2}{L}^{2}+{c}_{3}L+{c}_{4}+\left({c}_{5}{L}^{3}+{c}_{6}{L}^{2}+{c}_{7}L+{c}_{8}\right){\text{cos}}(\theta +{c}_{9})$$

**Figure 8:**
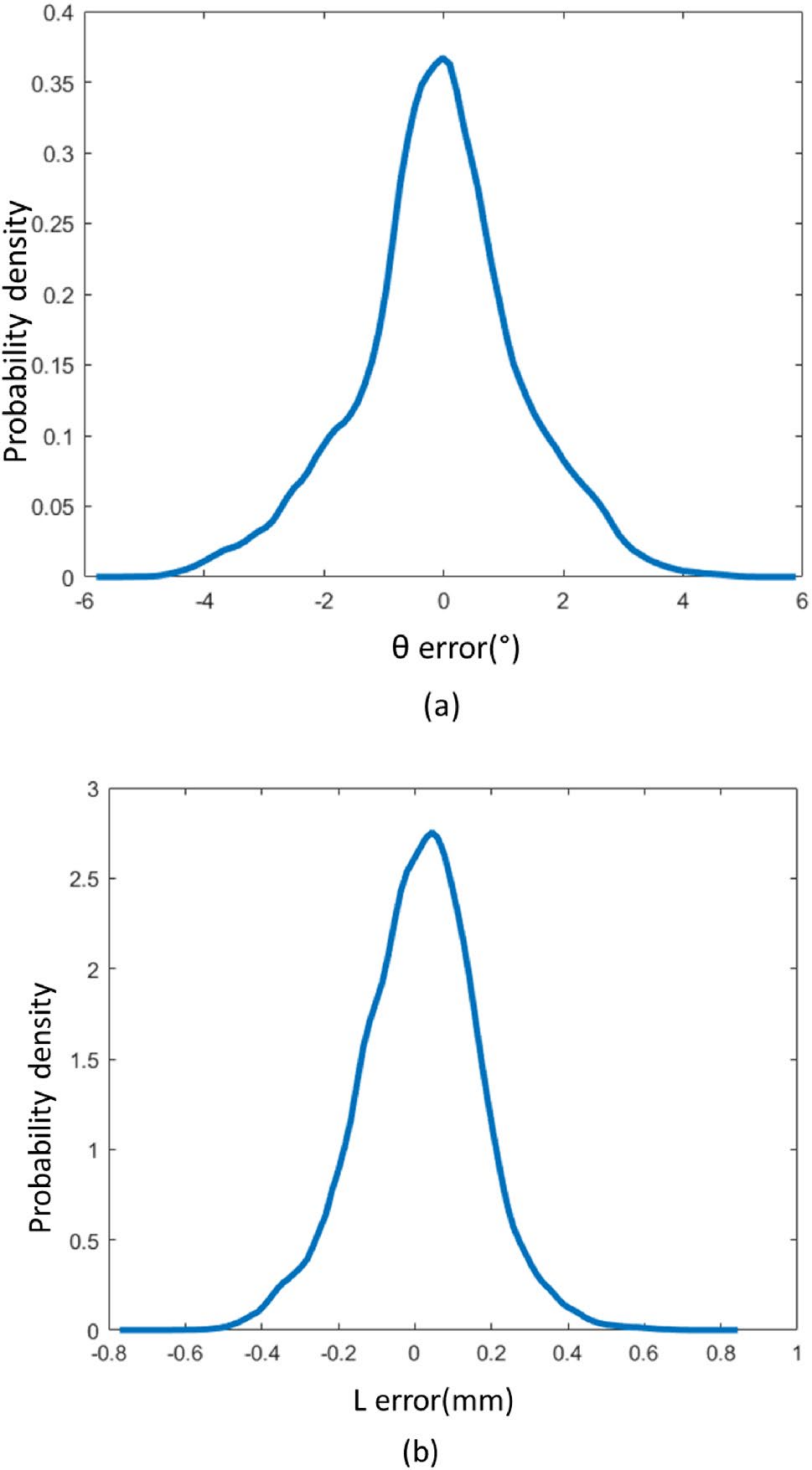
Error probability density obtained from mathematic modeling (**a**) rotary measurement error, (**b**) linear measurement error.

Subsequently, four equations were derived for each of the *R*s. This non-linear system of equations was solved using the ‘fsolve’ command in Matlab. The initial guess, which is the position of the last computed point, was considered as an input to the ‘fsolve’ command. The error probability density resulting from this method for 435 K points of the working region is depicted in Fig. 8. The accuracy and the Root Mean Square Error (RMSE) of this method are provided in (Table 2).

ML method, in comparison to MM, yields higher accuracy and computation speed at the cost of higher RAM occupation. The ML model occupies 4 gigabytes of RAM space, and it takes around two milliseconds to compute each displacement using a dual-core Core i7 processor with a maximum frequency of 3.5 GHz. MM method, on the other hand, takes around 6 ms for the same task.

Also, due to the necessity of providing an initial guess to the ‘fsolve’ function, the accuracy of the predictions is directly affected by this value. In this paper, the last calculated value is given as an initial guess to this function. Meanwhile, the MM method does not require any initial value.

### Hysteresis

The hysteresis property is one of the parameters that considerably impacts the mechanical behavior of hyper-elastic materials. Although this effect may not disrupt the performance of soft actuators, it can have detrimental effects on the efficiency of soft sensors, compelling us to use computational methods to eliminate it. In the sensor presented in this research, due to the interactions of two SPSCs placed in a set, hysteresis may cause changes in pressure during reciprocating movements. For this reason, the effects of hysteresis on longitudinal and rotational measurements have been experimentally investigated in this section.

**Figure 9:**
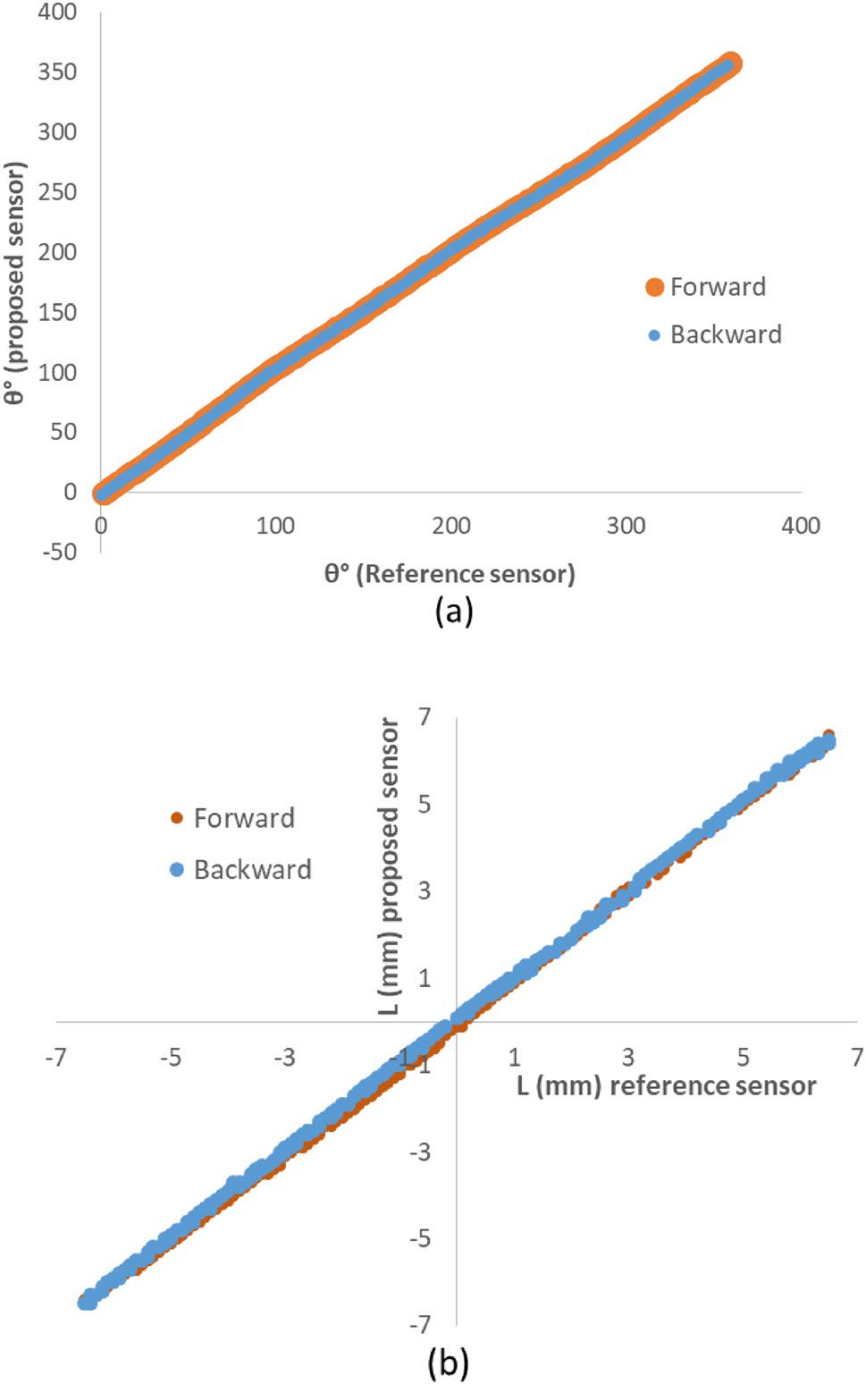
Hysteresis analysis diagrams (**a**) θ value in forward and backward rotary movements, (**b**) L value in forward and backward linear movements.

As shown in the Fig. 9, although this effect was not observed in the rotary direction, in linear displacements, a maximum of 0.2 mm hysteresis was observed.

### Resolution

**Figure 10:**
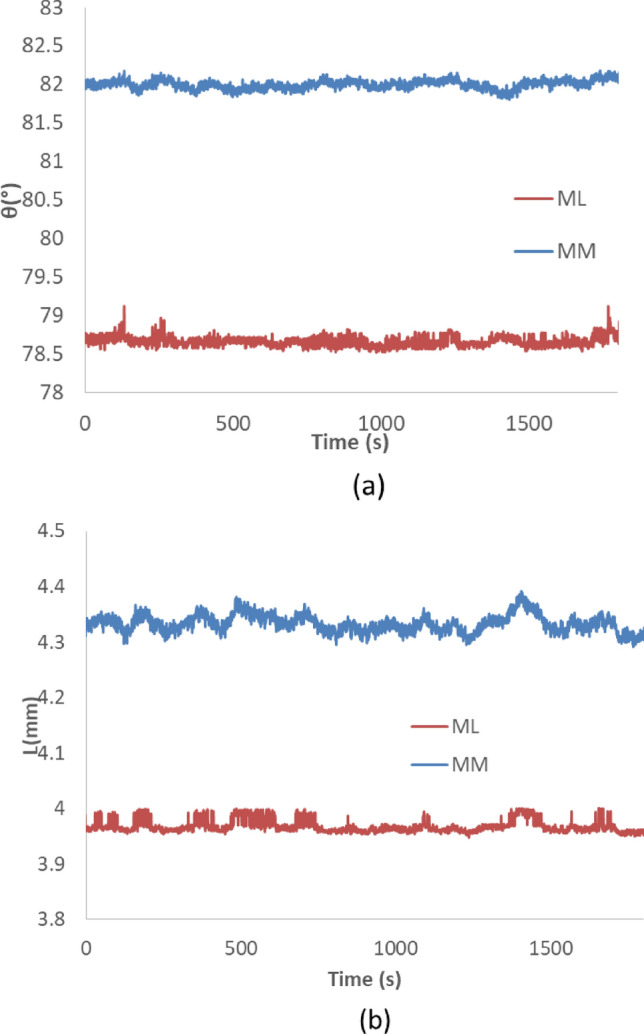
Measured values of stationary sensor during 30 min for ML and MM methods (**a**) rotary measurement, (**b**) linear measurement.

The output of the fabricated sensor by placing it in a state of rest for 30 min is shown in Fig. 10. The standard deviation of the data in this chart indicates the sensor resolution for two modes of rotary and linear measurements, which are mentioned in Table 2^27^. These values are more affected by the noise present in the data, the source of which is the noise on the pressure and temperature data of the BMP280 sensors. MM method is more susceptible to noise compared to ML method in linear measurements. Their performance is the same in rotary measurements.

### Dynamic response

**Figure 11:**
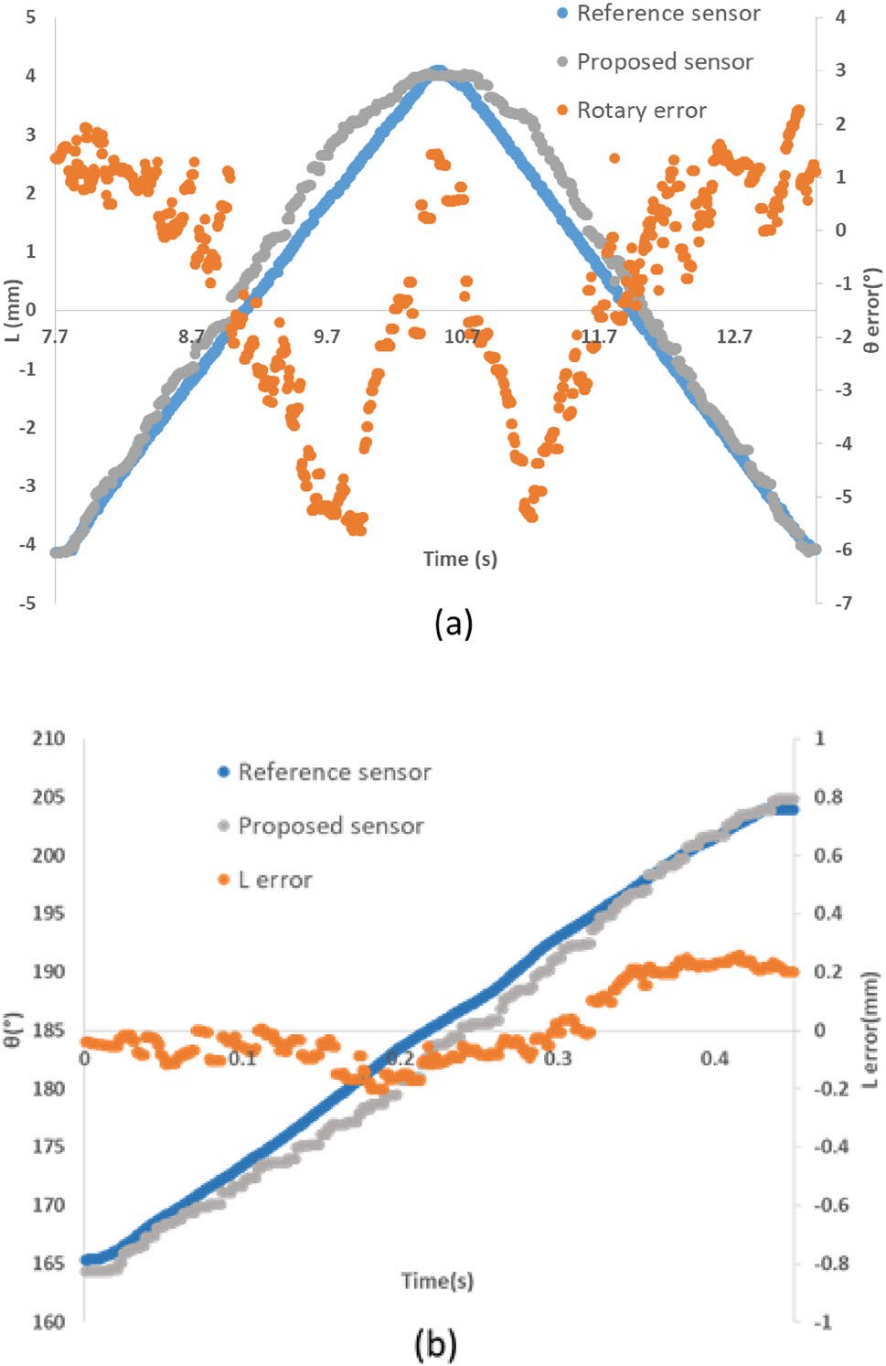
Dynamic response of sensor (**a**) linear response of the sensor at 3mm/s and its associated rotary error, (**b**) rotary response of the sensor at 15 RPM and its associated linear error.

The dynamic response of the sensor was evaluated up to a linear and rotational speed of 3 mm/s and 15 rpm with MM model. The graph obtained from this test at these two speeds and the resulting numerical values are shown in the Fig. 11, Tables 3 and 4. According to Fig. 11, dynamic displacements in one direction will increase the measurement error in the other direction as well. These errors can be derived from the speed of data acquisition and the nature of pneumatic measurement.

**Table 3:** Dynamic linear characteristics of the prototype sensor.

Linear speed (mm/s)	0.1	0.5	1	2	3
Error (mm)	0.25	0.32	0.37	0.48	0.8

**Table 4:** Dynamic rotary characteristics of the prototype sensor.

Rotary speed (RPM)	1	5	10	15
Error (^∘^)	2.8	3.1	3.9	5.2

### Discussion

A comparison of the performance characteristics of the proposed sensor with others is delineated in Table 5.

**Table 5:** Comparison between the presented sensor and the others.

Measurement method	Capacitive^12^	Optic^1^	Magnetic^3^	Triboelectric^13^	This work
Linear range (mm)	90	1000	50	70	13
Linear accuracy (mm)	0.81	–	$17\times {10}^{-3}$	–	0.49
Rotary range (°)	0–360	± 0.4	0–16	0–360	0–360
Rotary accuracy (°)	2.8	$4\times {10}^{-3}$	–	–	0.54
Linear resolution (mm)	$41\times {10}^{-3}$	$0.7\times {10}^{-3}$	$10\times {10}^{-3}$	2	$11\times {10}^{-3}$
Rotary resolution (°)	$0.15$	$0.8\times {10}^{-3}$	$5.7\times {10}^{-3}$	15	$0.06$
Volume $({{\text{cm}}}^{3})$	> 700	–	–	$\approx 700$	140
Electromagnetic compatibility	**	**	*	**	*****
Electric isolation	**	****	*	**	*****
Low-cost	***	**	**	**	*****

Due to the all-polymer structure, this sensor is fully compatible with electromagnetic environments. Other methods are not at all suitable for use in these environments due to metal mechanisms and the nature of electrical measurement. The magnetic sensor has the lowest score due to more interference with these waves. On the other hand, due to the use of air as an interface in the measurements and the significant distance between the data acquisition unit and the sensor, it is electrically isolated from the measured object. This feature is established in optical sensors due to the distance between the source of emission and light reception with the object to be measured, but the applied fields in capacitive, triboelectric, and magnetic methods are an obstacle to isolation. The measurement method presented in this article is very economical due to the polymer structure and the type of pressure sensors used. However, other methods require more complex circuits and data acquisition equipment.

## Conclusion

In conclusion, this study detailed the inception, design, testing, and assessment of the first soft sensor devised for the simultaneous measurement of linear and rotational displacements. The sensor was assembled using a series of SPSCs attached to a shaft. The efficacy of this sensor was appraised utilizing a setup tailored for linear and rotational displacements and calibration methodologies encompassing mathematical modelling and machine learning. The ML approach with random forest algorithm yielded superior outcomes compared to the alternative technique. As a result, within the linear movement range of ± 6.5 mm and rotational range of (0°–360°), the sensor's accuracy was ascertained to be 0.49 mm and 5.4°, respectively. By adjusting parameters such as tube length, eccentricity, initial contraction (C), and initial linear deflection (${D}_{0}$), the sensor could be designed according to the intended application.

This sensor embodies attributes that are unattainable in analogous measurement methods, such as:

1. No Electro-Magnetic interface (EMI) noise and the absence of electromagnetic wave emission. Additionally, the sensor lacks metal components, classifying it as an Electro-Magnetic Compatible (EMC) sensor.
2. Compact size due to simultaneous measurement of displacements and eliminating the movement mechanisms.
3. The capability to measure purely rotational movements without interference from concurrent linear movements along the L direction of the measured object. This is particularly advantageous in rehabilitation applications, as it minimizes movement constraints for individuals, thereby enhancing their comfort. This capability is also applicable in purely linear movement measurement scenarios, eliminating the need to restrict the object's rotational movements. This feature mitigates the stresses exerted on the sensor and actuator in industrial applications.
4. A more streamlined manufacturing process and cost-effectiveness compared to similar models.

## Data Availability

The code and dataset are available on: https://drive.google.com/drive/folders/1GX4ZJbJ_x1p9qLXqKUzTtiqSEdK7uUaM?usp=sharing.
